# Placental Growth Factor Expression Is Required for Bone Marrow Endothelial Cell Support of Primitive Murine Hematopoietic Cells

**DOI:** 10.1371/journal.pone.0067861

**Published:** 2013-08-02

**Authors:** Xiaoying Zhou, Lora W. Barsky, Gregor B. Adams

**Affiliations:** Eli and Edythe Broad Center for Regenerative Medicine and Stem Cell Research at USC, Keck School of Medicine, University of Southern California, Los Angeles, California, United States of America; Emory University, United States of America

## Abstract

Two distinct microenvironmental niches that regulate hematopoietic stem/progenitor cell physiology in the adult bone marrow have been proposed; the endosteal and the vascular niche. While extensive studies have been performed relating to molecular interactions in the endosteal niche, the mechanisms that regulate hematopoietic stem/progenitor cell interaction with bone marrow endothelial cells are less well defined. Here we demonstrate that endothelial cells derived from the bone marrow supported hematopoietic stem/progenitor cells to a higher degree than other endothelial or stromal cell populations. This support was dependant upon placental growth factor expression, as genetic knockdown of mRNA levels reduced the ability of endothelial cells to support hematopoietic stem/progenitor cells in vitro. Furthermore, using an in vivo model of recovery from radiation induced myelosuppression, we demonstrate that bone marrow endothelial cells were able to augment the recovery of the hematopoietic stem/progenitor cells. However, this effect was diminished when the same cells with reduced placental growth factor expression were administered, possibly owing to a reduced homing of the cells to the bone marrow vasculature. Our data suggest that placental growth factor elaborated from bone marrow endothelial cells mediates the regulatory effects of the vascular niche on hematopoietic stem/progenitor cell physiology.

## Introduction

Hematopoietic stem cells (HSCs) are maintained, and their physiology regulated, in specialized microenvironments known as the stem cell niche [1]. In the adult bone marrow (BM) two different stem cell niches have been proposed; the endosteal niche, where the osteoblasts are believed to maintain the quiescence and promote self-renewal of HSCs [2]–[4], and the vascular niche, where cells of the endothelial lineage or perivascular cells support the HSCs [5]. While many studies have been performed that examined the molecular and cellular interactions between the stem cells and the endosteal niche cells, little is know regarding the interactions between the stem cells and the cell types that comprise the vascular niche.

It has been shown that 60% of HSCs in the adult BM are in contact with sinusoidal endothelium, while only 14% are at the endosteal surface [6]. However, it is not known if direct contact with endothelial cells (ECs) in the vascular niche is required for self-renewal of HSCs as the mechanisms for the support remain relatively unknown. Previous studies examined the ability of primary adult mice ECs from non-hematopoietic organs such as heart, brain, liver, lung and kidney to support hematopoietic stem/progenitor cells (HSPC). Using in vitro co-culture assays as well as in vivo competitive repopulation assays, these studies demonstrated differences in the supportive ability of the ECs, as brain and heart ECs could expand the HSC population, while lung and liver ECs maintained the hematopoietic cells. However, the mechanism of support was not addressed [7]. Bis, an anti-apoptotic and stress response protein, has been identified as an important protein for the vascular niche with Bis^−/−^ mice demonstrating a defect in sinusoidal endothelium, as well as a loss of stromal cells expressing CXCL-12 or IL-7 [8]. Yet, the specific mechanisms directly influencing the HSCs are not known. Similarly, pleiotrophin (PTN) has been proposed as a secreted component of the BM vascular niche as PTN^−/−^ mice demonstrated a reduction in BM HSCs [9]. But these effects were only correlated with an expression of PTN in BM ECs.

Recently, a functional regulatory effect of ECs on HSCs has been reported [10]. Here, a primary human EC line expressing the adenoviral E4ORF1 gene could promote self-renewal of murine LT-HSCs in vitro which could thus augment BM repopulation in vivo. The mechanism of action was related to the Notch pathway as Notch ligand expression on the BMECs promoted expansion of LT-HSCs in vivo. The relevance of these studies to the in vivo setting is unknown as the ECs were of human origin and the support of murine HSCs was investigated. However, the authors have recently further expanded these observations to demonstrate that human CD34^+^ cells co-cultured on these ECs are able to expand their in vivo repopulation potential compared to cells cultured in cytokines alone [11]. More recent investigations into the mechanisms of support of primitive HSCs by ECs have came from Ding and colleagues who specifically deleted stem cell factor (SCF) from various proposed components of the niche and examined the effects on the primitive cells [12]. Here, they showed that SCF expression from ECs is essential for HSC function, while deletion of expression from other stromal cell types in the BM does not affect the primitive hematopoietic cells. Similar studies from the same group, as well as another independent group, have also found that deletion of CXCL12 from endothelial cells led to a specific decline in HSC number or function in the adult BM [13], [14]. However, in all of these studies, direct interactions between the HSCs and the ECs themselves were not addressed.

We wished to determine which specific cells of the murine BM vascular niche plays a predominant role in controlling HSPC physiology, and the mechanism by which the vascular niche may directly exert its effects. Previous studies have suggested that HSPCs isolated from different regions of the BM microenvironment have different biological potentials [15], [16]. We hypothesized that niche-related elements contributed to these effects and therefore examined whether the ECs comprising the vascular niche derived from different regions of the BM have differing effects on the regulation of HSPCs. We demonstrated that central marrow ECs showed enhanced support of HSPC growth in vitro. This correlated with expression of placental growth factor (PlGF), a factor which has been shown to be essential for the recovery of the hematopoietic system following BM myelosuppression [17]. Using genetic knockdown strategies, we demonstrate a role for PlGF in the support of primitive cells in vitro. Further, by using an in vivo model of recovery from radiation induced myelosuppression, we demonstrate that by transplanting central marrow EC to sublethally irradiated mice, the recovery from myelosuppression was accelerated. However this effect was abrogated upon administration of PlGF knockdown central marrow ECs.

## Materials and Methods

### Animals

6 to 8 week-old male C57Bl/6 and Balb/c mice (Taconic Farms Inc, Oxnard, CA) were obtained and used in accordance with the University of Southern California (USC) Institutional Animal Care and Use Committee (IACUC) guidelines. These studies were approved by the USC IACUC, Protocol # 11601. USC is fully accredited by the Association for the Assessment and Accreditation of Laboratory Animal Care, International (AAALAC; Assurance # A3518–01). Mice were housed in sterilized microisolator cages and received autoclaved food and water *ad libitum*. They were monitored daily for signs of distress and measures and sacrificed at the study end points using CO_2_ delivered by compressed gas followed by cervical dislocation.

### Isolation of murine BM cells

C57Bl/6 mice were sacrificed by CO_2_ asphyxiation and cervical dislocation, and the femora and tibia were dissected. To prepare the central marrow mononuclear cells (MNCs), the BM from the femora and tibia were flushed in Minimum Essential Medium, Alpha (α-MEM) supplemented with 10% fetal bovine serum (FBS; all from Mediatech Inc.) using a 1 ml syringe with 25-gauge needle (BD Biosciences). To prepare the endosteal marrow cells, the marrow-depleted femora and tibia were cut into 1–2 mm diameter fragments and crushed in α-MEM with 10% FBS using a mortar and pestle and incubated at 37°C with a type I collagenase (3 mg/mL; Worthington) in α-MEM with 10% FBS for 90 minutes at 37°C. The cell suspensions were filtered through a 70-μm cell strainer (BD Biosciences).

### Culture conditions of ECs and stromal cells (SCs) from murine BM cells

BM MNCs from the central marrow and endosteal marrow were plated on rat plasma vitronectin (Sigma-Aldrich) coated 25 cm^2^ flasks (BD Biosciences) at a density of 1×10^6^ cells/cm^2^ in EC basal medium (EBM-2) supplemented with a cytokine cocktail (SingleQuots; Clonetics) containing 5% FBS, as previously reported [18]–[20]. For cultivation of SCs, BM MNCs from the central marrow and endosteal marrow were plated on 25 cm^2^ flasks at a concentration of 1×10^6^ cells/cm^2^ in long term culture medium (M5300; StemCell Technologies). The cultures of ECs or SCs were replaced with new media every 3 days for 10 days.

### Fluorescence immunocytochemistry

ECs or SCs were plated in 4-well plates (BD Biosciences) and fixed with cold paraformaldehyde (4%) for 15 minutes. The cells were then incubated with blocking buffer, followed by rat anti-mouse CD31 (BD Pharmingen) and goat anti-mouse VE-cadherin (R&D Systems) overnight at 4°C. Secondary FITC-labeled antibodies (goat anti rat IgG, Rabbit anti goat IgG; Sigma Aldrich) were then added for 1 hour at room temperature. Cells were mounted in Vectashield medium with DAPI (Vector Lab) to preserve fluorescence and counterstain nuclei.

### Alkaline phosphatase (ALP) staining

ECs or SCs were plated in 24-well plates and stained for ALP activity according to the manufacture's instructions (Takara, MK300).

### *In vitro* DiI-Ac-LDL labeling

ECs were plated in 24-well plates and incubated with 10 µg/ml DiI-ac-LDL (Invitrogen) for 4 hours at 37°C, washed four times with PBS and then fixed with paraformaldehyde (4%). Cells were then mounted in Vectashield with DAPI.

### *In vitro* capillary tube formation

3×10^4^ ECs were harvested in 200 μl EBM-2 medium supplemented with the cytokine cocktail and 50 ng/ml VEGF (PeproTech Inc), and were plated in 48-well plate coated with 150 μl Matrigel (BD Biosciences) for 1 hour at 37°C. Capillary tube formation on Matrigel was observed after 5–16 hours of incubation.

### Lentivirus production and transduction into the BM ECs

Small inhibitory hairpin RNAs (shRNA) sequences targeting PlGF were purchased from Open Biosystems. The packaging plasmid psPAX2 and the envelope plasmid pMD2G were a kind gift of Dr. Wange Lu. Recombinant lentiviruses were individually produced by transient transfection of 293T cells using calcium phosphate transfection. Viral supernatants were collected 48 and 72 hours after transfection and passed through a 0.45 µm filter (Nalgene). For EC transductions, viral supernatant was added when the ECs reached about 70%–80% confluent. A non-targeting shRNA sequence (GFP shRNA) was used as a control. Drug selection with puromycin (8 μg/ml) was initiated 24 hour after transduction, and the culture was then maintained under drug selection conditions.

### Quantitative reverse transcription–polymerase chain reaction

Total RNA was extracted using the RNA Miniprep Kit (Stratagene) and reverse-transcribed into cDNA using the SuperScript VILO cDNA synthesis kit (Invitrogen) in accordance with the manufacturer's instructions. To quantify the expression of Tie-2, VE-Cadherin, vascular endothelial growth factor a (VEGFa), vascular endothelial growth factor b (VEGFb) and PlGF, Taqman Gene Expression Assay primers and probe sets (Applied Biosystems and Roche Diagnostics) were used. Expression of HPRT served as an endogenous control. Levels of gene expression were quantified with using the 7900HT real-time polymerase chain reaction system (Applied Biosystems). Standard curves were created with the use of QPCR mouse reference total RNA (Stratagene).

### Flow cytometric analysis

ECs or SCs were harvested from culture flasks using cell dissociation solution (Sigma) treatment, and stained with the EC-specific markers anti-mouse CD31, Flk-1, Sca-1 and the hematopoietic cell marker anti-mouse CD45 (all from eBioscience). The stained cells were filtered through 30 µm nylon mesh, and then the cell populations in the ECs or SCs culture condition were determined. For measuring the frequency of HSPCs in the BM, the BM MNCs from Balb/c mice were incubated in phosphate-buffered saline (PBS; Mediatech Inc) with phycoerythrin (PE) conjugated anti mouse Sca-1, PE-cyanine 7 (PE-Cy7) cojugated anti mouse c-kit, fluorescein isothiocyanate (FITC) conjugated anti mouse CD48 and allophycocyanin (APC) conjugated anti mouse CD150. Concurrently, cells were incubated with a biotin lineage cocktail. After primary antibody incubation, cells were stained with PE-cyanine 5 (PE-Cy5) streptavidin (All from BD Biosciences). Cell frequencies were measured on an LSR II flow cytometer (Becton Dickinson).

### Flow cytometric sorting

MNCs were obtained as described above. Cells were then incubated in PBS (Mediatech Inc) with peridinin chlorophyll protein (PerCP) anti mouse CD45, phycoerythrin (PE) anti mouse CD31, and biotin anti-mouse TER-119. After primary antibody incubation, cells were stained with Allophycocyanin-Cy7 (APC-Cy7) streptavidin (All from BD Biosciences). The endothelial cells and stromal cells were determined as CD45^-^TER-119^−^CD31^+^ and CD45^−^TER-119^−^CD31^−^ respectively. To obtain the hematopoietic stem and progenitor cells, the bone marrow MNCs were stained with PE anti mouse Sca-1, PE-cyanine 7 (PE-Cy7) anti mouse c-kit and a biotin lineage cocktail (CD3e, CD11b, B220, Gr-1, and TER-119). After primary antibody incubation, cells were stained with peridinin chlorophyll protein (PercP) streptavidin (All from BD Biosciences). The stained cells were sorted with the use of a FACSAria™ flow cytometer (Becton Dickinson) on the basis of established cell surface phenotype.

### Co-culture of LSK cells

ECs and SCs were harvested, irradiated at 15Gy, and plated in 12-well plates (2×10^5^ cells per well) in serum free medium. BM LSK cells were then added at 1×10^3^ cells per well and maintained at 37°C/5% CO_2_. After 7 days, nonadherent cells were harvested by washing the monolayers gently with warm PBS three times. The number of hematopoietic cells was measured as CD45^+^ cells by flow cytometry. To analyze the number of CFU-Cs, 1×10^2^ LSK cells per well were co-cultured with the irradiated ECs or SCs in 12-well plates. The nonadherent cells were collected, and then suspended in MethoCult GF M3434 (StemCell Technologies) and cultured at 37°C/5% CO_2_ in a humidified atmosphere. The number of CFU-Cs was scored on day 8 according to standard criteria.

### Cobblestone area-forming cell (CAFC) assay

BM MNCs or purified LSK cells were seeded in serial dilutions on a confluent layer of central marrow ECs, central marrow SCs, spleen ECs, PlGF knock down ECs and mock transduced ECs which were previously irradiated at 15Gy, or OP9 stromal cells that were previously irradiated at 35 Gy. Cells were maintained in α-MEM supplemented with 10%FBS at 33°C in a 5% CO_2_ incubator with weekly half-medium changes. The presence of cobblestone areas [21] was scored on day14, 28, 35, 42 and 49. The frequency of CAFCs was calculated with the use of L-Calc™ software (StemCell Technologies).

### *In vivo* homing and lodgment

The cultured ECs, PlGF knock down ECs, mock transduced ECs and the cultured SCs, were harvested and labeled with carboxyfluorescein diacetate succinimidyl ester (CFDA-SE; Invitrogen) according to the manufacturer's instructions. The labeled cells were injected into the tail vein of the Balb/c mice that were irradiated at 550cGy approximately 4 hours before transplantation on day 0, and then injected to mice intraperitoneally on day 1 to 4 (1×10^6^ cells per mouse per day). The mice were then sacrificed on day 5, and the frequency of labeled cells was measured by the detection of CFDA-SE^+^ cells in the BM and spleen by flow cytometry. To assess the lodgment of injected cells to bone marrow, the tibias from the Balb/c mice were dissected on day 5 after irradiation, decalcified for 3 days in Immunocal (Decal Chemical Corporation), and embedded in paraffin blocks after processing. 5 µm serial sections of tibia were cut and mounted with Vectashield containing 4,6-diamidino-2-phenylindole (DAPI; Vector Laboratories).

### Immunohistochemistry

For detection of VE-cadherin, paraffin sections of femur were antigen-retrieved using Target Retrieval Solution (DAKO, CA) followed by endogenous peroxidase (3% H_2_O_2_) and nonspecific protein block (5% BSA, 10% donkey serum, and 0.02% Tween-20), then incubated with goat anti-mouse VE-cadherin (R&D Systems) overnight at 4°C. Slides were incubated with secondary antibody biotin conjugated donkey anti-goat IgG (Jackson IR) for 1 hour and streptavidin horseradish peroxidase (Jackson IR) for 30 minutes at room temperature, and then were developed with DAB staining kit (DAKO) for 5 minutes at room temperature and counterstained in Mayer's hematoxylin. A cover-slip was applied to the slides with the use of the Cytoseal XYL mounting medium (Richard-Allan Scientific). The light phase image was collected using Zeiss Axio Imager Z1 and Zeiss Axio observer A1 fluorescence microscope with Axiovision Rel 4.8 software (Carl Zeiss).

### Statistical analysis

Comparison of experimental groups was performed using the paired or unpaired two-tailed Student's *t*-test as appropriate for the dataset. p<0.05 was considered significant.

## Results

### Characterization of endothelial and stromal cell cultures

**Figure 1:**
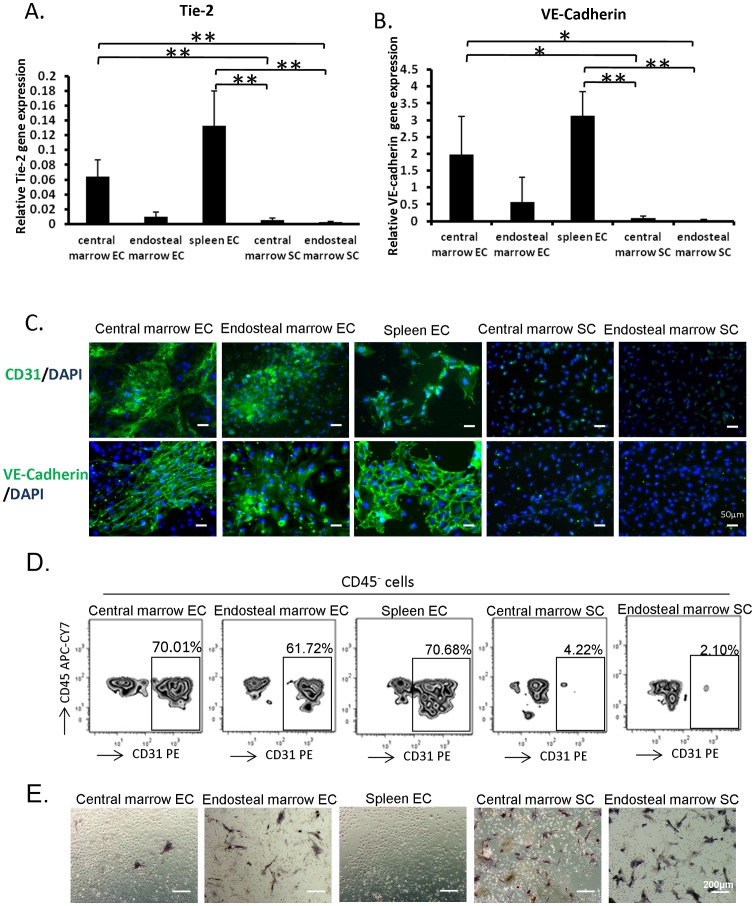
Characteristics of cells grown in endothelial or stromal cell culture conditions. (A, B) Total RNA was extracted from central marrow EC, endosteal marrow EC, spleen EC, central marrow SC and endosteal marrow SC. The mRNA expression level of Tie-2 and VE-Cadherin was measured using quantitative RT-PCR. The relative expression was normalized to Hypoxanthine-guanine phosphoribosyltransferase (HPRT) levels and calculated from standard curves. (*p<0.05, **p<0.01; n = 6 from 3 independent experiments; error bars represent standard deviation). (C) Cells cultured in endothelial or stromal culture conditions were stained with antibodies to CD31 and VE-Cadherin. Positive signals were visualized with FITC conjugated secondary antibody. Nuclei were visualized with 4,6-diamidino-2-phenylindole (DAPI). (D) Central marrow EC, endosteal marrow EC, spleen EC, central marrow SC and endosteal marrow SC were harvested by enzyme-free cell dissociation solution. The expression of CD31 and CD45 was analyzed by flow cytometry with PE conjugated anti-CD31 and APC-Cy7 conjugated anti-CD45. (E) Cells cultured in endothelial or stromal culture conditions were stained with alkaline phosphatase activity. Positive reaction was observed as dark blue violet in the cells.

BM MNCs were isolated from central marrow or endosteal marrow, and grown in either endothelial or stromal cell culture conditions. ECs from the spleen were also cultured as a comparison with the BM derived ECs. In these experiments, the five types of cultured cells; central marrow derived ECs, endosteal marrow derived ECs, spleen derived ECs, central marrow derived SCs and endosteal marrow derived SCs, were characterized for endothelial specific markers. The cells in endothelial culture conditions presented typical endothelial cobblestone morphology after reaching confluence, while the cells in the stromal culture conditions showed spindle-shape cells and lacked the cobblestone morphology (data not shown). The mRNA expression level of endothelial related genes, Tie-2 [22] and VE-cadherin [23], were significantly higher in the central marrow derived ECs and spleen ECs, while the expression in endosteal marrow ECs was only marginally greater than either stromal cell culture (Figure 1A, B). To further examine the endothelial phenotype, endothelial specific markers including CD31 and VE-cadherin [24] were evaluated in the cultured cells by fluorescence immunocytochemistry and flow cytometry. Cells grown in all EC culture conditions expressed CD31 and VE-cadherin, but the central marrow SCs and endosteal marrow SCs did not (Figure 1C). The percentage of expression of CD31 or VE-cadherin in the ECs was about 40%–50%. When the ECs were detached from the culture flasks using a cell dissociation solution treatment instead of the trypsin-EDTA [25], a much higher level of ECs was detected in EC cultures derived from central marrow, endosteal marrow and spleen as compared to central marrow SCs and endosteal marrow SCs (Figure 1D). The cells in the ECs culture condition also had the ability to take up DiI-Ac-LDL and form capillary-like tubes in Matrigel. To evaluate the composition of non-endothelial cells in the stromal and endothelial culture conditions, we assessed the level of osteoblasts. Osteoblasts are one of the components of primary bone marrow stromal cells [26], and characterized by the expression of alkaline phosphatase activity [27]. We observed no, or very few, ALP^+^ cells in the central marrow and spleen derived ECs cultures. However, the cells in the endosteal marrow derived ECs culture condition contained about 10% ALP positive cells. The higher proportion of ALP^+^ cells (40%–50%) were observed in the central marrow and endosteal marrow derived SCs culture condition as compared to the cells in the ECs culture condition (Figure 1E). Therefore, highly EC enriched primary cultures were used in these experiments.

### Central marrow ECs demonstrated enhanced support of primitive hematopoietic cells

**Figure 2:**
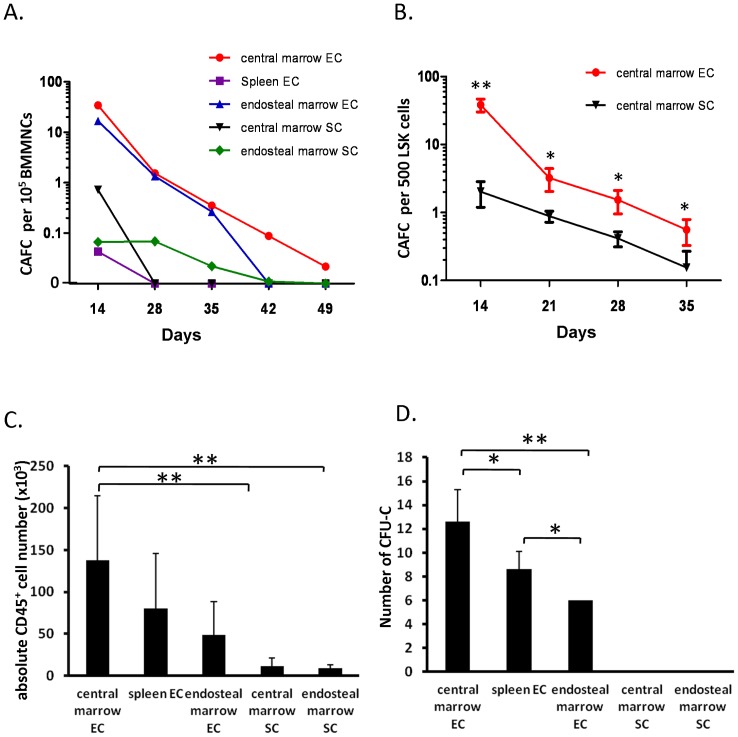
Central marrow ECs demonstrate enhance support of primitive hematopoietic cells. (A) BM MNCs were seeded in serial dilutions on central marrow EC, central marrow SC, endosteal marrow EC, endosteal marrow SC and spleen EC and cultured at 33°C and 5% CO_2_. CAFCs were scored on day 14, 28, 35, 42 and 49. (B) BM LSK cells were seeded in serial dilutions on central marrow EC and central marrow SC and cultured at 33°C and 5% CO2. CAFCs were scored on day 14, 21, 28 and 35 (**p<0.01, *p<0.05 n = 4 from 3 independent experiments; error bars represent standard deviation). (C) 1000 LSK cells were co-cultured with the five supportive cell layers and the number of CD45^+^ cells on day 7 was assessed by flow cytometry (**P<0.01; n = 5 from 2 independent experiments; error bars represent standard deviation). (D) 100 LSK cells were co-cultured with the five supportive cell layers and the number of CFU-Cs was assessed on day 7 (*P<0.05, **P<0.01; n = 5 from 2 independent experiments; error bars represent standard deviation).

We next assessed the ability of the five types of cultured cells to maintain primitive hematopoietic cells *in vitro* using the functional CAFC assay. The CAFC frequency at shorter (days 14) and longer (days 28–49) time intervals was used to measure relatively mature and primitive hematopoietic cells, respectively. As shown in Figure 2A, the frequencies of short-term (day 14) and long-term (day 35) CAFCs of BM MNCs on central marrow EC and endosteal marrow EC were higher compared with those on spleen EC, central marrow SC or endosteal marrow SC. The CAFC frequencies of BM MNCs on central marrow EC and endosteal marrow EC from day 14 to day 35 were similar, but these two curves diverged at culture times after day 35. On day 49 of culture, CAFCs were detectable only on central marrow ECs. We further compared the effects of central marrow ECs and central marrow SCs to support the primitive hematopoietic cell activity of purified lineage^-^Sca-1^+^c-kit^+^ (LSK) cells. The CAFC activity in the LSK cells subpopulation on the central marrow ECs were significantly higher than that on the central marrow SCs at day 14 and day 35 (Figure 2B). These data indicate that central marrow or endosteal marrow derived ECs have enhanced support of primitive hematopoietic cell growth over spleen derived ECs and BM derived SCs. In addition, central marrow ECs are capable of maintaining primitive hematopoietic cell activity for longer time intervals over the other supportive layers.

In order to determine the effect of the five types of cultured cells on the proliferation and differentiation of primitive hematopoietic cells, we measured the number of CD45^+^ hematopoietic cells and CFU-C activity, following a 7 day co-culture of LSK cells. We found that the central marrow ECs could promote the proliferation of CD45^+^ hematopoietic cells by 2 to 15 fold higher than other types of supportive cells (Figure 2C). In addition, the generation of CFU-Cs on central marrow ECs was significantly higher than that on other supportive layers (Figure 2D). These results demonstrated that central marrow ECs are superior in their ability of promoting proliferation and differentiation of primitive hematopoietic cells than other feeder layers. Interestingly, spleen ECs were able to support the proliferation and differentiation of the primitive hematopoietic cells (Figure 2C, D), but were not able to support more primitive CAFCs (Figure 2A).

### PlGF expression is specifically higher in the central marrow ECs

**Figure 3:**
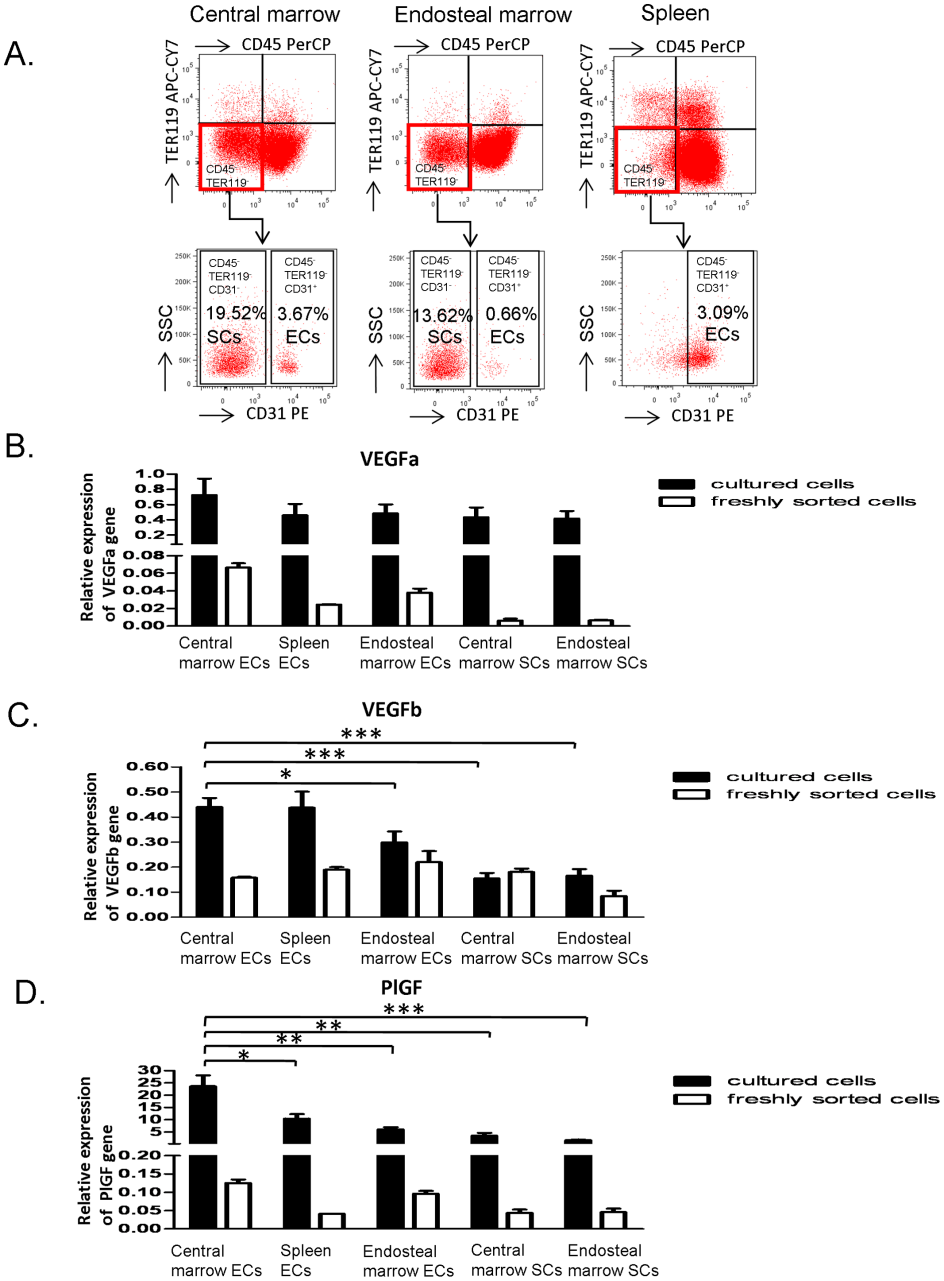
mRNA expression of VEGFa, VEGFb and PlGF in cultured and fresh ECs and SCs. Total RNA was extracted from the cultured and freshly isolated central marrow EC, endosteal marrow EC, spleen EC, central marrow SC and endosteal marrow SC. (A) Flow cytometric plots for the purification of ECs and SCs derived from central marrow, endosteal marrow and spleen. SCs and ECs were identified as CD45^−^TER119^−^CD31^−^ and CD45^−^TER119^−^CD31^+^ respectively. The mRNA expression level of VEGFa (B), VEGFb (C) and PlGF (D) was measured using quantitative RT-PCR. The relative expression was normalized to *hprt* levels and calculated from standard curves (For the cultured cells, *p<0.05, **p<0.01, ***p<0.001; n = 6 from 3 independent experiments; error bars represent standard deviation. For the freshly sorted cells, n = 2).

Recent studies have suggested that VEGF-A and PlGF may be important cytokines involved in the reciprocal interaction between primitive hematopoietic cells and ECs [17], [28]–[32]. Therefore, we postulated that VEGF-family factors secreted from ECs may act upon the primitive hematopoietic cells to regulate their physiology. To examine the mechanism of enhanced support of primitive hematopoietic cells by the central marrow ECs, we performed real-time PCR analysis for the expression of VEGFa, VEGFb and PlGF. The five types of cultured cells expressed VEGFa at very similar levels (Figure 3B). VEGFb expression in cultured central marrow ECs was higher than cultured endosteal marrow ECs, cultured central marrow SCs and cultured endosteal marrow SCs, but the expression was similar to cultured spleen ECs (Figure 3C). In contrast, the expression of PlGF was significantly higher specifically in the cultured central marrow ECs compared with the other types of cultured cells (Figure 3D). In addition, we examined the expression of VEGFa, VEGFb and PlGF in freshly isolated central marrow ECs, endosteal marrow ECs, spleen ECs, central marrow SCs and endosteal marrow SCs (Figure 3A). Albeit at a much lower level, in contrast to the cultured cells, VEGFa expression in freshly sorted ECs derived from central marrow, spleen and endosteal marrow was higher than the central marrow SCs and endosteal marrow SCs (Figure 3B), whereas the expression of VEGFb in freshly sorted ECs and SCs was similar (Figure 3C). However, correlating with the cultured cells, the expression of PlGF in the freshly sorted central marrow ECs and endosteal marrow ECs was higher than the spleen ECs, central marrow SCs and endosteal marrow SCs (Figure 3D). These results suggest that PlGF was predominantly expressed on endothelial cells but not on stromal cells in the mouse BM. Therefore, due to this high expression in the cultured central marrow ECs, we hypothesized that PlGF may mediate the supportive role of these cells on primitive hematopoietic cells.

### Reduced PlGF expression in central marrow ECs diminishes their ability to support primitive hematopoietic cells

**Figure 4:**
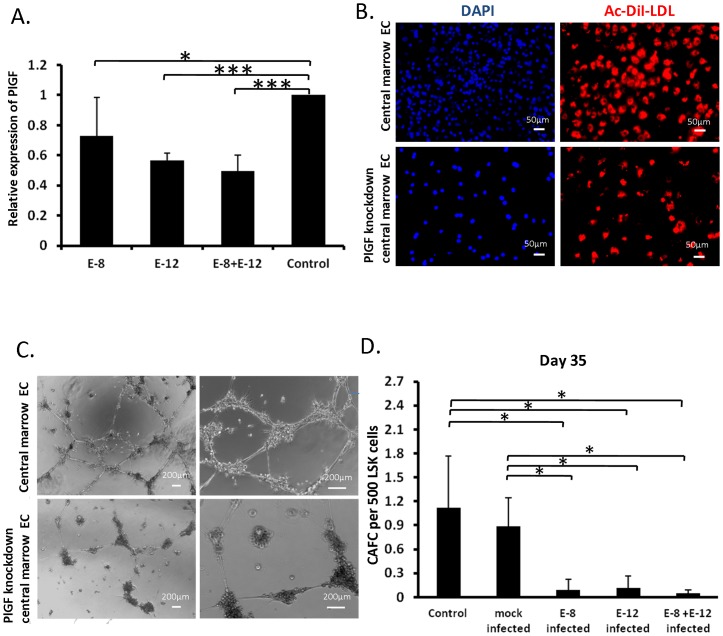
Down-regulation of PlGF in central marrow ECs diminishes their ability to support primitive hematopoietic cells. (A) Total RNA was obtained from central marrow ECs transduced with shRNA clones targeting PlGF. The mRNA expression level of PlGF was measured using quantitative RT-PCR and normalized to *hprt* levels (*p<0.05, ***p<0.001; n = 6 from 3 independent experiments; error bars represent standard deviation). (B) Uptake of DiI-Ac-LDL (red) in control-transduced and PlGF knockdown central marrow ECs. Nuclei were visualized with DAPI (Scale bar: 50 µm). (C) The formation of endothelial capillary like tube was evaluated under a phase-contrast microscope on Matrigel following 10 hours of culture (Scale bar: 200 μm). (D, E) LSK cells were seeded in serial dilutions on central marrow ECs, central marrow ECs transduced with E8, E12 or E8 and E12 lentiviral shRNA-PlGF vectors or shRNA-GFP vector and cultured at 33°C and 5% CO_2_. CAFCs were scored on week 2 and 5 (*p<0.05, **p<0.01; n = 3 from 3 independent experiments; error bars represent standard deviation).

To determine whether the increased expression of PlGF plays a functional role in the support of the primitive hematopoietic cells, we used a genetic ‘knockdown’ approach to reduce PlGF gene expression in the cultured central marrow ECs. Anti-PlGF shRNA vectors were transduced into central marrow ECs and PlGF expression was quantified using quantitative RT-PCR. We found that among five different shRNA vectors, E8 and E12, as well as their combination, significantly reduced PlGF expression compared to control cells (Figure 4A). To exclude the possibility that reduction of PlGF grossly altered the properties of the ECs, we examined the characteristics of ECs before and after PlGF knockdown. We found that PlGF knockdown central marrow EC took up Ac-LDL uniformly as well as non-transduced central marrow EC (Figure 4B). Additionally, PlGF knockdown central marrow EC formed capillary-like tubules within 5–16 hours when plated on Matrigel (Figure 4C). Analysis of the average length of the endothelial tubules demonstrated that there was no significant difference between the control and the PlGF knockdown cells (278 μm ^+^/−50 µm versus 267 μm ^+^/−94 μm, respectively). These results demonstrated that PlGF knockdown central marrow EC retained endothelial characteristics. To directly evaluate the ability of these cells to support primitive hematopoietic cells *in vitro*, LSK cells were seeded on central marrow ECs transduced with E8, E12 and E8+E12. We found that there was an significant inhibition of long term (day 35) CAFCs activity on PlGF shRNA transduced central marrow ECs compared with the mock transduced central marrow ECs and non-transduced central marrow ECs (Figure 4D). These results suggested that PlGF is involved in the supportive effect of central marrow ECs on primitive hematopoietic cells in vitro.

### Knockdown of PlGF expression in central marrow ECs inhibits recovery of primitive hematopoietic cells *in vivo*

**Figure 5:**
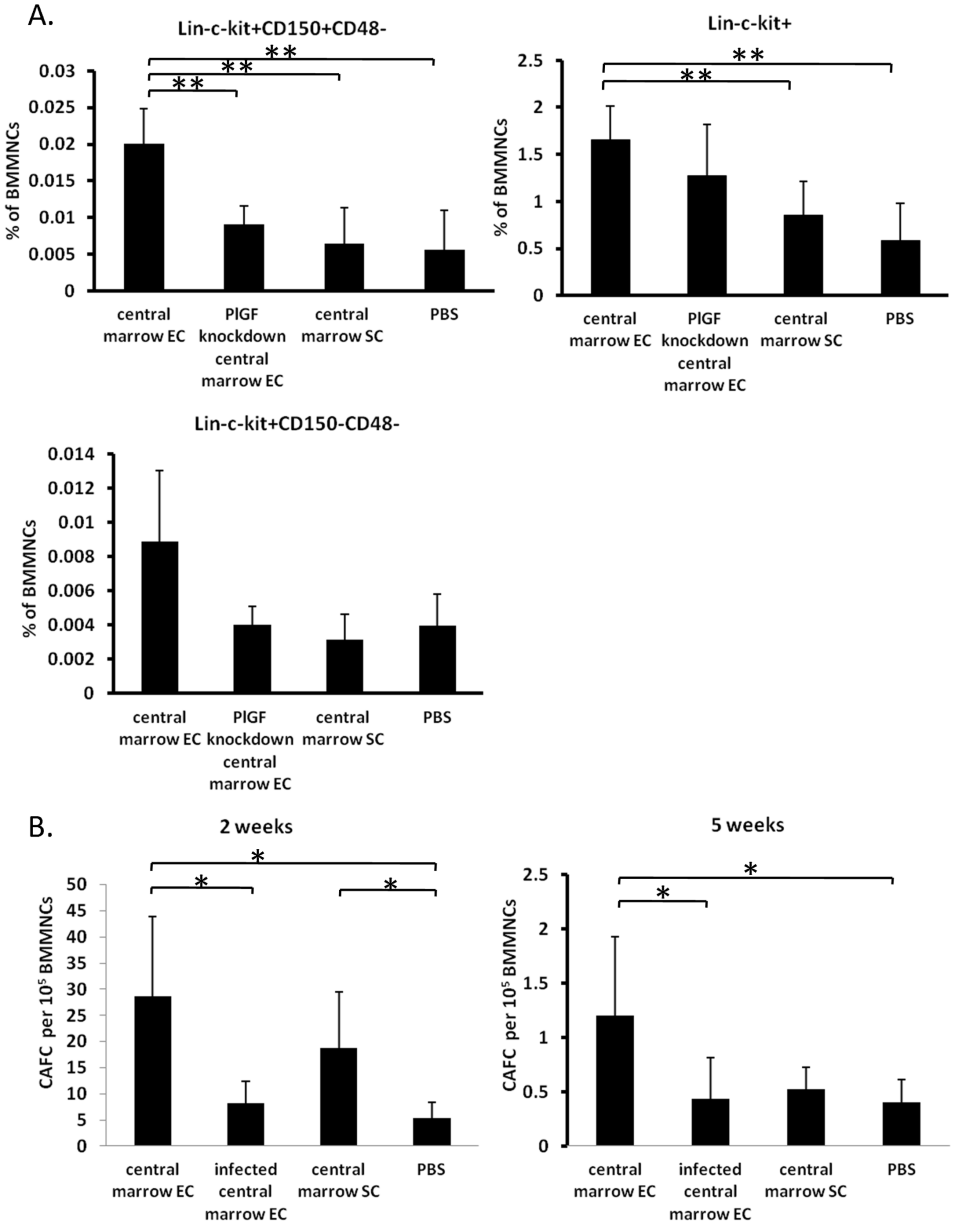
Reduced PlGF expression diminishes hematopoietic recovery *in vivo*. (A) BM MNCs from Balb/c mice following treatment with central marrow ECs, PlGF knockdown central marrow ECs, central marrow SCs or PBS were evaluated for LT-HSC, ST-HSC and HPC frequencies using flow cytometry (**p<0.01; n = 9 from 3 independent experiments; error bars represent standard deviation). (B) BM MNCs from the same mice were assessed for CAFC frequencies at weeks 2 and 5 (*p<0.05; n = 9 from 3 independent experiments; error bars represent standard deviation).

Previous studies have demonstrated that following sub-lethal irradiation, mice injected with murine fetal blood ECs displayed accelerated recovery of hematopoiesis with essentially normal BM sinusoid vessel architecture recovery by day 20 following irradiation [33], [34]. To investigate whether PlGF is involved in this process, we transplanted central marrow ECs, PlGF knockdown central marrow ECs, central marrow SCs or PBS into Balb/c mice that were irradiated with 550cGy and evaluated the recovery of the hematopoietic system at day 20 post-irradiation. Sca-1 is not informative as an HSPC marker in Balb/c mice [35], therefore this was excluded from the analysis of the primitive hematopoietic cells. We identified Lin^−^c-Kit^+^CD150^+^CD48^−^ cells as LT-HSCs, Lin^−^c-Kit^+^CD150^−^CD48^−^ cells as ST-HSCs and Lin^−^c-Kit^+^ cells as hematopoietic progenitor cells (HPCs). The frequency of LT-HSCs was significantly increased in the central marrow EC treated mice, but not PlGF knockdown EC or central marrow SC treated mice compared to PBS treated controls. Similarly, the frequency of HPCs was significantly increased in the central marrow EC treated mice, but not in PlGF knockdown EC or central marrow SC treated mice (Figure 5A). The frequency of ST-HSCs in the central marrow EC treated mice suggested an increase, but was not significantly different (Figure 5A). To assess the function of the primitive hematopoietic cells, we performed CAFC assays. We found that the frequency of day 14 and day 35 CAFCs from central marrow EC treated mice were significantly higher than that from PlGF knockdown central marrow EC or PBS treated mice (Figure 5B). The frequency of CAFC in central marrow stromal cells demonstrated an increase at day 14, but no difference at day 35 compared with PBS treated mice (Figure 5B). These results demonstrated that, similar to previous reports [33], the administration of central marrow ECs were able to accelerate the recovery of hematopoiesis, however reduction of PlGF expression in the same cells abrogated this effect.

### Knockdown of PlGF expression in central marrow ECs inhibits the recovery of BM vasculature *in vivo*

**Figure 6:**
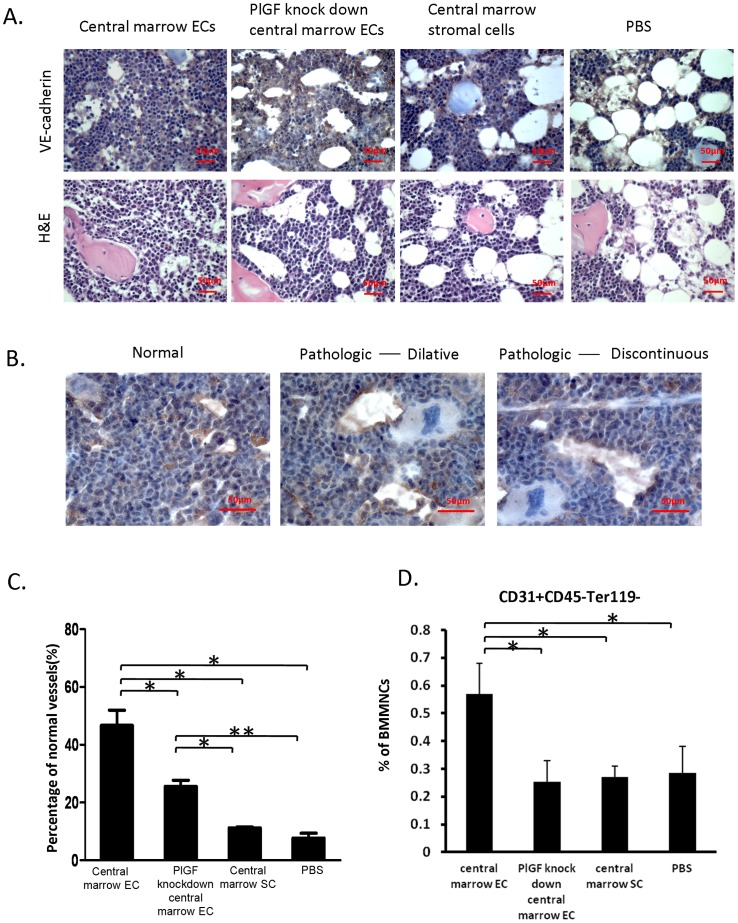
Reduced PlGF expression diminishes vascular recovery *in vivo*. (A) Central marrow ECs, PlGF knockdown central marrow ECs, central marrow SCs or PBS were transplanted into Balb/c mice following 550cGy total body irradiation. On day 20 post irradiation, the tibias were harvested and assessed by immunohistochemistry for VE-cadherin expression (scale bar: 50 μm). (B) Representative images of normal and pathologic vessels (scale bar: 50 μm). (C) Quantitation of normal or pathologic vessels in the BM of the irradiated mice transplanted with the different cell types. Vessels were counted in three 200X fields per section. (*p<0.05, **p<0.01; 40 sections per mouse, n = 9 from 3 independent experiments). (D) BM MNCs from the treated mice were harvested and stained with the antibody to CD31, CD45 and Ter-119. The percentage of BM ECs was determined by CD31^+^CD45^−^Ter119^−^ cells using flow cytometric analysis (*p<0.05; n = 9 from 3 independent experiments; error bars represent standard deviation).

In the Balb/c mice that were treated with central marrow ECs, PlGF knockdown central marrow ECs, central marrow SCs and PBS following irradiation at 550cGy, we observed a regression of BM sinusoid vessels by H&E and anti-VE-cadherin immunohistochemical staining (Figure 6A). To characterize the degree of regenerating sinusoid vessels, we quantified morphologically normal vessels and pathologic vessels, presenting as dilated or discontinuous vessels based on the VE-Cadherin^+^ BM ECs (Figure 6B). We observed a predominance of pathologic vessels in PBS, central marrow SC and PlGF knockdown central marrow EC treated mice. However, in central marrow EC treated mice there was significant vascular recovery (Figure 6C). Furthermore, we examined the frequency of ECs in BM, defined as CD31^+^CD45^−^Ter119^−^, and found that the frequency of these cells in the BM of central marrow EC treated mice was significantly higher than the other treated mice (Figure 6D). These results indicated that the administration of central marrow ECs were able to augment the replenishment of BM vasculature. However, reduction of PlGF expression in the same cells disturbed this effect.

### Transplanted central marrow ECs contributed to the BM vasculature recovery in irradiated mice

**Figure 7:**
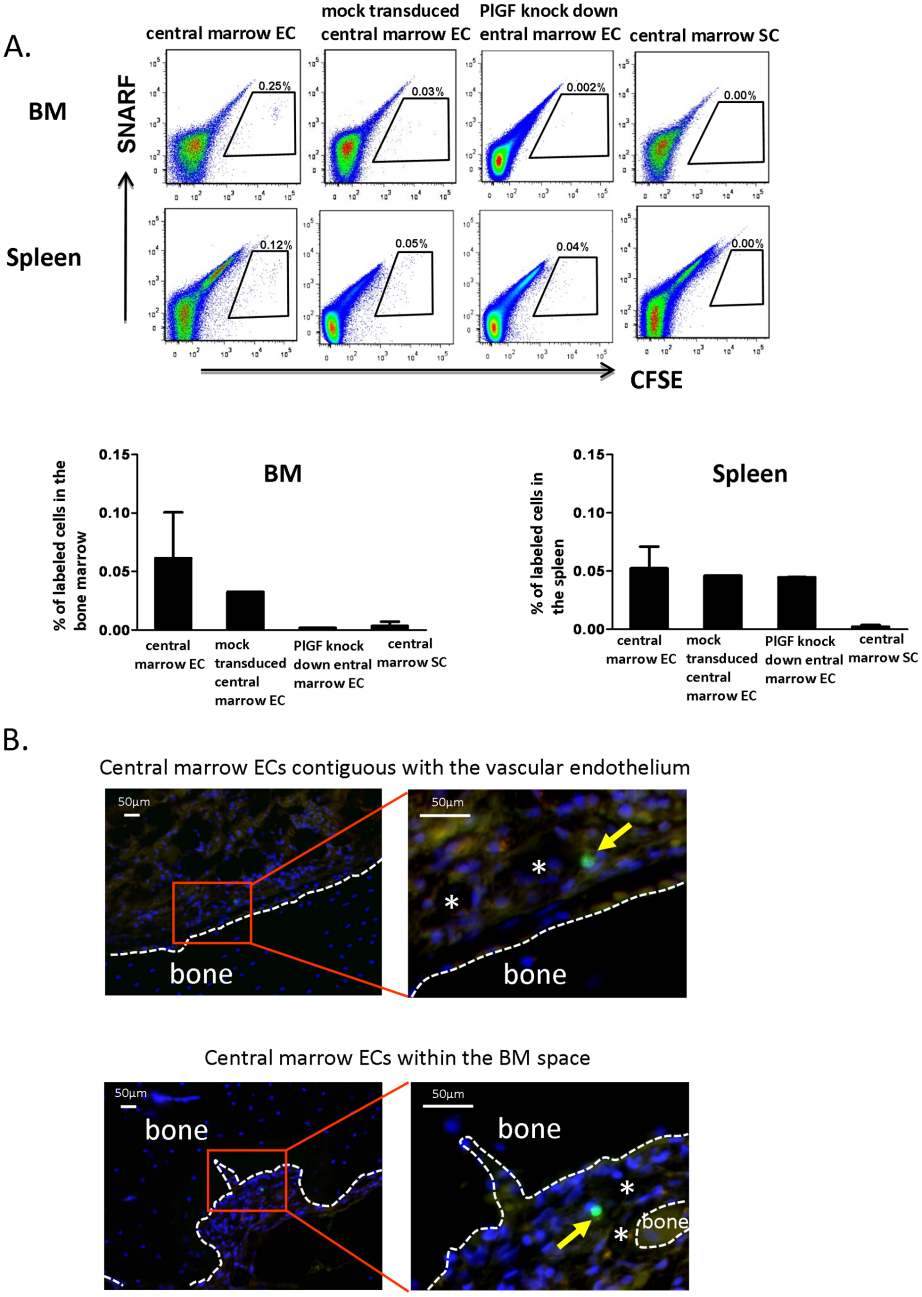
Transplanted central marrow ECs incorporate into the adult BM vasculature. (A) Central marrow ECs, PlGF knockdown central marrow ECs, mock transduced central marrow ECs or central marrow SCs were labeled with CFDA-SE and transplanted into Balb/c mice following 550cGy total body irradiation (1×10^6^ cells per mouse; intravenous injection on day 0 and intraperitoneal injection on day 1 to day 4). The percentage of labeled cells present in the BM and spleen on day 5 post irradiation was determined by flow cytometry. Representative flow plots (left) and percentage of labeled cells present in the BM and spleen (right) are shown (for central marrow ECs and SCs, n = 6 from 2 independent experiments; for PlGF knockdown central marrow ECs and mock transduced central marrow ECs, n = 2). (B) Lodgment of the central marrow ECs in the BM, classified as contiguous with the vascular endothelium (top image) or within the BM space (bottom image). The BM vessel is indicated by an asterix. Bone is outlined by the dashed line. The yellow arrows represent the transplanted central marrow EC labeled with CFDA-SE. Nuclei were visualized with DAPI (blue) present in Vectashield (scale bar: 50 μm; 40 sections per mouse, n = 6 from 2 independent experiments).

To determine whether the transplanted central marrow ECs were directly incorporated into the reconstituting vasculature of the BM, central marrow ECs and SCs were labeled with CFDA-SE and injected into the irradiated mice. Our results demonstrate that the central marrow ECs had a greater frequency of homing to BM and spleen compared with central marrow SCs as assessed by flow cytometry (Figure 7A). A reduced homing level of PlGF knockdown central marrow ECs in BM, but similar homing level in spleen, was detected compared to the mock transduced central marrow ECs (Figure 7A). With respect to lodgment of the central marrow ECs, histologic assessment of the tibias showed that the 37.5% CFDA-SE^+^ cells were contiguous with the vascular endothelium (Figure 7B top), while 62.5% CFDA-SE^+^ cells were within the BM space (Figure 7B bottom). CFDA-SE^+^ cells were not detected in the BM of the mice treated with PlGF knockdown central marrow ECs or central marrow SCs (data not shown). Therefore, these results demonstrate that the cultured central marrow ECs were capable of homing to BM and lodging in the BM vascular niche, which presumably led to augmented recovery of hematopoiesis and the vasculature in BM of the irradiated mice. While the PlGF knockdown central marrow ECs were able to home to the spleen similar to the controls, they had a specific defect in homing to the vasculature structures of the BM. Therefore, PlGF may play a role in the recovery of hematopoiesis through multiple different mechanisms involving direct effects of the HSPCs, as well as interactions with other BMECs. These studies thus highlight the challenge of separating direct effects of ECs on the primitive hematopoietic cells and on the vasculature, which may in turn affect the HSPCs.

## Discussion

Initial studies of the HSC niche focused on the endosteal and vascular niche as being distinct entities, with the endosteal niche supporting the LT-HSCs, while the vascular niche supports ST-HSCs [36], [37]. More recent data has suggested that they may be indistinct and that both the osteoblasts and EC play key roles, potentially in the same niche [38]. However, the question still remains as to what role the EC is playing in the niche and what are the molecular signals by which it exerts its effects. In addition, while cells of the osteoblast lineage are specifically localized to the endosteal surface, ECs are found in different regions of the BM. Whether these ECs in different regions of the BM differentially regulate HSPCs has not been explored.

ECs are known to release angiocrine factors, such as growth factors or chemokines, which promote organogenesis and cell differentiation [39]–[42]. These angiocrine factors are also known to affect hematopoiesis. Recently, it has been reported that angiocrine expression of the Notch ligands Jagged 1 and Jagged 2 on human ECs is necessary for the support of LT-HSCs [10], [11]. VEGF is known to regulate HSC repopulation and survival [29], [30], as well as coordinate with Angiopoietin-1 (Ang-1) to induce mobilization of HSCs by temporal and regional activation of VEGF/VEGFR2 and Ang-1/Tie-2 signaling pathways [43]. In addition, PlGF promoted recruitment of VEGFR1^+^ HSCs from a quiescent to a proliferative BM microenvironment, favoring differentiation, mobilization and reconstitution of hematopoiesis [17].

We compared the effects of ECs or SCs isolated from the endosteal or central region of the BM, or the spleen on the support of growth of HSPCs in vitro to mimic the complexity of the BM microenvironment *in vivo*. We demonstrated that the ECs from the BM were superior in their ability to maintain primitive hematopoietic cells, with the central marrow ECs showing enhanced support at longer time intervals. In addition, central marrow ECs supported increased proliferation and differentiation of progenitor cells. We also found that the expression of PlGF was significantly increased in the central marrow ECs compared to other supportive cells. This effect of central marrow ECs on supporting primitive hematopoietic cells growth in vitro was diminished when expression of PlGF was knocked down. Hence, we proposed that secreted PlGF from BM ECs may influence the physiology of HSCs, although we could not rule out an autocrine action on the ECs themselves as well.

While it has been shown that osteoblasts are key constituents of the HSC niche under homeostatic conditions, it has been proposed that they do not play a role in hematopoietic reconstitution after myeloablation, rather this is the role of the ECs [44]. In our in vivo model of recovery from radiation induced myeloablation, different cell populations were transplanted into sublethally irradiated mice. It has been shown that transplanted ECs do not engraft in the BM [45], however, peripheral blood [46], [47], bone marrow [18]–[20], liver [48], [49] or lung [50] derived ECs have also been isolated in vitro and the transplanted ECs could restore impaired neovascularization in a hind limb ischemia [47], [51] or myocardial ischemia [52], [53] model. In our study the transplanted BM ECs were able to home and lodge in the BM, either at or adjacent to the vasculature. They also had a positive effect on the recovery of hematopoiesis as evidenced by an increased frequency of primitive hematopoietic cells. These effects were specific for ECs, since the transplanted BM SCs were only able to induce HPC recovery, as assessed by the week 2 CAFC assay, and were not detected in BM. Correlating with the in vitro assays, knockdown of PlGF expression in the BM ECs reduced the effect down to no cell treatment levels. However, these effects may be simply due to a lack of homing of the cells specifically to the BM vasculature. This highlights a complicating factor in these studies, which is that the effect of PlGF was to also stimulate normal sinusoid vessel recovery, potentially through the specific migration of the ECs to the bone marrow endothelium. Therefore, as is the case in many studies of the vascular niche, separating direct effects on the primitive hematopoietic cells from effects on the vasculature which may then further promote the reconstitution of the HSPCs becomes challenging.

Taken together, our data demonstrate that ECs in the BM vascular niche are involved in the regeneration of HSPCs and that PlGF plays a role in this overall process. Whether or not this mechanism is also involved in the regulation of HSC self-renewal and quiescence at the endosteal surface was not addressed, but it is possible that different mechanisms may occur here. Future studies may be required to delineate the potential role of different HSC niches in the bone marrow and how they regulate hematopoiesis as a whole.
